# Longitudinal Associations of Adherence to the World Cancer Research Fund/American Institute for Cancer Research (WCRF/AICR) Lifestyle Recommendations with Quality of Life and Symptoms in Colorectal Cancer Survivors up to 24 Months Post-Treatment

**DOI:** 10.3390/cancers14020417

**Published:** 2022-01-14

**Authors:** Marlou-Floor Kenkhuis, Floortje Mols, Eline H. van Roekel, José J. L. Breedveld-Peters, Stéphanie O. Breukink, Maryska L. G. Janssen-Heijnen, Eric T. P. Keulen, Fränzel J. B. van Duijnhoven, Matty P. Weijenberg, Martijn J. L. Bours

**Affiliations:** 1 Department of Epidemiology, GROW School for Oncology and Developmental Biology, Maastricht University, 6211 LK Maastricht, The Netherlands; eline.vanroekel@maastrichtuniversity.nl (E.H.v.R.); jose.breedveld@maastrichtuniversity.nl (J.J.L.B.-P.); mjanssenheijnen@viecuri.nl (M.L.G.J.-H.); mp.weijenberg@maastrichtuniversity.nl (M.P.W.); m.bours@maastrichtuniversity.nl (M.J.L.B.); 2 Department of Medical and Clinical Psychology, Tilburg University, 5037 AB Tilburg, The Netherlands; f.mols@uvt.nl; 3 Department of Surgery, GROW School for Oncology and Developmental Biology, NUTRIM School of Nutrition and Translational Research in Metabolism, Maastricht University Medical Centre+, 6229 HX Maastricht, The Netherlands; s.breukink@mumc.nl; 4 Department of Clinical Epidemiology, Viecuri Medical Center, 5912 BL Venlo, The Netherlands; 5 Department of Internal Medicine and Gastroenterology, Zuyderland Medical Centre Sittard-Geleen, 6162 BG Geleen, The Netherlands; e.keulen@zuyderland.nl; 6 Division of Human Nutrition and Health, Wageningen University & Research, 6708 PB Wageningen, The Netherlands; franzel.vanduijnhoven@wur.nl

**Keywords:** colorectal cancer survivorship, lifestyle recommendations, health-related quality of life, fatigue, chemotherapy-induced peripheral neuropathy

## Abstract

**Simple Summary:**

Using data from 459 colorectal cancer (CRC) survivors, we described how participants adhered to the World Cancer Research Fund/American Institute for Cancer Research (WCRF/AICR) lifestyle recommendations and how this adherence was related to quality of life, level of functioning, symptoms of fatigue, and neuropathy symptoms. We found that increases in a lifestyle score was associated with better physical functioning and less fatigue. No relations with neuropathy symptoms were found. In addition, we observed that physical activity played an important role in the lifestyle score with regards to quality of life. In contrast, we observed that body composition and alcohol recommendations had a counteractive influence within the lifestyle score. Our findings suggest that CRC survivors benefit from overall adherence to the WCRF/AICR lifestyle recommendations in terms of quality of life and fatigue. Specific recommendations have a varying influence on these associations, complicating the interpretation and requiring further study.

**Abstract:**

Post-treatment adherence to the World Cancer Research Fund/American Institute for Cancer Research (WCRF/AICR) lifestyle recommendations were associated with health-related quality of life (HRQoL), fatigue, and chemotherapy-induced peripheral neuropathy (CIPN) in colorectal cancer (CRC) survivors. In a prospective cohort among CRC survivors (*n* = 459), repeated home-visits were performed at 6 weeks, 6, 12, and 24 months post-treatment. Dietary intake, body composition, sedentary behaviour, and physical activity were assessed to construct a lifestyle score based on adherence to seven 2018 WCRF/AICR recommendations. Longitudinal associations of the lifestyle score with HRQoL, fatigue, and CIPN were analysed by confounder-adjusted linear mixed models. A higher lifestyle score was associated with better physical functioning and less activity-related fatigue, but not with CIPN. Adjustment for physical activity substantially attenuated observed associations, indicating its importance in the lifestyle score with regards to HRQoL. In contrast, adjustment for body composition and alcohol inflated observed associations, indicating that both recommendations had a counteractive influence within the lifestyle score. Our findings suggest that CRC survivors benefit from an overall adherence to the WCRF/AICR lifestyle recommendations in terms of HRQoL and fatigue, but not CIPN. Specific recommendations have a varying influence on these associations, complicating the interpretation and requiring further study.

## 1. Introduction

Advances in early detection and treatment, combined with the aging of, and an increase in, the global population, have resulted in a marked rise in the number of individuals living with a past diagnosis of colorectal cancer (CRC) [1,2,3]. The post-treatment period is demanding for CRC survivors, mainly due to the major mental and physical health implications that accompany the cancer diagnosis and recovery of treatment, which consequently affect health-related quality of life (HRQoL) [4,5,6]. Two commonly experienced problems are cancer-related fatigue and chemotherapy-induced peripheral neuropathy (CIPN). In the long term (>5 years post-diagnosis), cancer-related fatigue is still experienced by one-third of CRC survivors [7]. Approximately two-thirds of patients experience CIPN symptoms, such as tingling hands and feet, after chemotherapy cessation, with falling rates to approximately one-third of patients at 6 months [8]. Both fatigue and CIPN can negatively affect physical functioning and daily living up to 10 years post-treatment [9,10,11]. The growing population of CRC survivors indicates the importance of targeting adverse effects of cancer treatment and HRQoL. It is also important to gain a better understanding of the relationships of these post-treatment health problems with lifestyle behaviours, including dietary habits and physical activity, since healthful changes in these behaviours could self-empower CRC survivors to improve their health, functioning, and quality of life.

In 2018, the World Cancer Research Fund and the American Institute for Cancer Research (WCRF/AICR) updated their lifestyle recommendations based on extensive systematic reviews and meta-analyses of evidence on diet, nutrition, physical activity, body weight, and risk of cancer [12]. As the evidence to formulate specific recommendations for cancer survivors is insufficient to date, WCRF/AICR advises them to follow the lifestyle recommendations for cancer prevention. These guidelines encourage cancer survivors to achieve and maintain a healthy lifestyle through weight management, regular physical activity, eating a healthy diet, and limiting alcohol consumption. These guidelines are associated with less recurrence and increased survival in cancer survivors, including CRC survivors [13,14,15,16]. In addition, adhering to the WCRF/AICR recommendations is likely also relevant for increasing HRQoL [17,18,19,20] as well as decreasing fatigue and CIPN [21].

Several studies have shown associations between individual lifestyle factors, such as physical activity, dietary habits, body mass index (BMI), and alcohol consumption, with HRQoL, fatigue, and CIPN in CRC survivors [19,20,22,23]. These findings have been mostly limited, however, by cross-sectional study designs. Recently, our research group investigated the role of individual WCRF/AICR recommendations in CRC survivors up to two years post-treatment. We found that increases in adipose tissue and muscle mass [24], moderate-to-vigorous physical activity (MVPA) [25], fruit, vegetable, and dietary fibre consumption [26], and decreases in sedentary behaviour [25], sugar-sweetened drink consumption, ultra-processed food (UPF) intake, and energy density [27] were independently and longitudinally associated with improved HRQoL and decreased fatigue, but not with CIPN. However, because the individual WCRF/AICR lifestyle recommendations form a package that, taken together, direct people towards healthy patterns of diet and physical activity, we aimed to further analyse the role of overall adherence to the updated lifestyle recommendations from the 2018 WCRF/AICR guidelines in this paper. By making use of the previously reported guidelines for operationalizing the level of adherence to the 2018 WCRF/AICR lifestyle recommendations [28], we aim to examine how the overall adherence in the form of a lifestyle score is longitudinally associated with HRQoL, fatigue and CIPN in CRC survivors from 6 weeks up to 2 years post-treatment.

## 2. Materials and Methods

### 2.1. Study Design and Population

Data analysis was performed with longitudinal data collected up until 16 July 2018, from the Energy for Life after ColoRectal cancer (EnCoRe) study. The EnCoRe study is an ongoing prospective cohort study for which patient recruitment began in 2012 at three participating hospitals in the south of The Netherlands. Inclusion criteria were men and women above the age of 18, diagnosed with stage I, II, or III CRC. Exclusion criteria were stage IV CRC, inability to understand and speak Dutch, residential address outside of the Netherlands, or the presence of comorbidities that could impede successful study participation, including cognitive and visibility/hearing disorders [29].

Patients were enrolled at diagnosis (*n* = 459) and followed up with repeated measurements at 6 weeks (*n* = 396), 6 months (*n* = 348), 12 months (*n* = 287), and 24 months (*n* = 208) post-treatment. The main reason for the decrease in numbers as follow-up time increases was due to participants with data available at diagnosis not having reached some of the subsequent follow-up points on 16 July 2018. Participation rate at diagnosis was 45% and follow-up participation rates were above 91% at all post-treatment follow-up timepoints. A detailed flowchart has previously been published by Kenkhuis and colleagues [24]. Study measurements were performed during home-visits by trained dietitians according to standard operating procedures. The Medical Ethics Committee of the University Hospital Maastricht and Maastricht University approved the study, and all patients provided informed consent.

### 2.2. Data Collection of the Lifestyle Score

Based on the level of adherence to the 2018 WCRF/AICR lifestyle recommendations for cancer prevention [12], we constructed a lifestyle score according to the operationalization proposed by Shams-White and colleagues [28]. We included seven WCRF/AICR lifestyle recommendations, including recommendations regarding body fatness (BMI and waist circumference), physical activity (MVPA and sedentary behaviour), and diet (ultra-processed foods, plant-based foods, meat consumption, and sugary and alcoholic drinks). We did not operationalise the breast-feeding recommendation as it was not relevant for this population. As suggested by Shams-White and colleagues, we did not operationalize the supplement recommendation. An overview of the applied WCRF/AICR lifestyle (sub)-recommendations, as well as the operationalisation of the level of adherence (no, partial, and complete) to the recommendations, and scoring thereof, are shown in Table 1.

The first WCRF/AICR recommendation is to limit body fatness [12]. Anthropometric measurements were performed by trained dietitians at patients’ homes to operationalise the body fatness recommendation. In order to compute BMI (kg/m^2^) at every timepoint, duplicate measurements of body height (cm) at diagnosis and of body weight (kg) at every time point was used. Waist circumference (cm) was measured in duplicate midway between the lowest rib and iliac crest at every time point, as another measure for body fatness.

The second WCRF/AICR recommendation is to be physically active as part of one’s daily life [12]. Hours/week of MVPA was determined using the validated Short QUestionnaire to ASsess Health-enhancing physical activity (SQUASH) questionnaire [30]. MVPA included all activities (walking, cycling, gardening, odd jobs, sports, household activities, and work) with a metabolic equivalent (MET) value ≥ 3. Accelerometers were used for obtaining objective data on time spent on sedentary behaviour, that is, activities during waking hours with low to very low energy expenditure (≤1.5 MET values, e.g., sitting, lying). Thigh-mounted tri-axial MOX activity monitors (MMOXX1; Maastricht Instruments B.V.) were worn by participants 24 h per day during 7 consecutive days, enabling accurate measurement of activities at low intensities and in different postures [31,32]. Prolonged sedentary behaviour (hours/day), which is sedentary time accrued in uninterrupted sedentary bouts with a duration of at least 30 min, was used in the scoring system [33]. Data were collected and processed as previously described [23].

WCRF/AICR recommendations three to seven, focussing on diet, are to limit the consumption of fast foods and sugary drinks; to eat mostly plant-based foods by having a higher intake of fruit, vegetables, and dietary fibre; to limit animal-based food products by reducing red and processed meat intake; and to limit the consumption of alcoholic drinks [12]. Structured dietary records were filled in by participants on 7 consecutive days at every post-treatment time point to obtain quantitative data on food intake. These dietary records were coded by trained dietitians. The 7-day mean daily intake of foods and drinks were available for data analyses. We made use of the Dutch Food Composition table (NEVO-2011) [34] as database for assessing the WCRF/AICR dietary recommendations, using dietary groups (e.g., for red meat, processed meat, sugary drinks, fruits, and vegetables) that existed or were specifically created with a web-based platform that connects details of intake with a nutrient database (Compl-eat, developed by Wageningen University & Research, Provincie Gelderland, The Netherlands). 

Each recommendation was assigned a score of 1 point (complete adherence), 0.5 point (partial adherence), and 0 points (non-adherence) to calculate the lifestyle score. Predefined cut-off values of the WCRF/AICR recommendations were used, except for the recommendation on fast food where cut-offs were based on tertiles. The consumption of fast foods was defined as UPF and classified according to the NOVA system that classifies food groups based on the extent of processing [28,35,36]. The fast foods score was calculated by using energy percent of UPF consumed relative to total energy intake (EN%), and, subsequently, participants were categorized based on tertiles. Three recommendations (healthy weight, physical activity, and a diet rich in wholegrains, vegetables, fruit, and beans) each included two sub-recommendations (see Table 1). For these sub-recommendations, a score of 0.5 points was assigned for complete adherence, 0.25 for partial adherence, and 0 for non-adherence. The recommendation score was the sum of each sub-recommendation score; possible scores were 0, 0.25, 0.5, 0.75, and 1. A lifestyle score for the WCRF/AICR recommendations was calculated by summing the scores of the seven operationalised recommendations (score range: 0–7). Higher adherence to the WCRF/AICR lifestyle recommendations was thus indicated by a higher score, reflecting a healthier lifestyle.

### 2.3. HRQoL, Fatigue and CIPN

At each post-treatment timepoint, the well-validated, cancer-specific European Organization for the Research and Treatment of Cancer Quality of Life Questionnaire-Core 30 (EORTC QLQ-C30) [37,38] was used to measure HRQoL outcomes, including global quality of life (QoL), physical, role and social functioning, and fatigue with. All scale scores were linearly transformed to a 0–100 scale, with higher scores on the functioning scales and global QoL reflecting better functioning or HRQoL, whereas higher symptom scale scores indicate more symptoms (i.e., worse fatigue). In addition, a summary score (SumSc) was calculated from the mean of 13 of the 15 subscores (excluding the financial difficulties and global QoL questions) according to recommendations by the EORTC Quality of Life Group [38,39].

Besides the fatigue symptom scale from the EORTC QLQ-C30, fatigue was also assessed by the validated 20-item Checklist Individual Strength (CIS) to enable a more comprehensive multidimensional assessment of fatigue [40,41]. The CIS consists of four subscales: subjective fatigue (range: 8–56), concentration problems (5–35), reduced motivation (4–28), and activity-related fatigue (3–21). Each item was scored on a 7-point Likert scale. The total fatigue score (range: 20–140) was obtained by summing all item scores. Higher scores represent more fatigue. We included subjective fatigue, activity-related fatigue, and the total fatigue score in the present analyses since we expected the lifestyle recommendations to be associated with the physical and subjective dimensions of fatigue. 

CIPN complaints were measured with the EORTC QLQ-CIPN20 at each home-visit. This 20-item questionnaire consists of sensory, motor and autonomic subscales, and a summary score [42]. All scale scores were linearly converted to a 0–100 scale [43], with higher scores indicating more CIPN symptoms.

### 2.4. Other Factors

Sociodemographic characteristics including age and sex, and clinical information on cancer stage, chemotherapy/radiotherapy, and tumour site were retrieved from medical records. Educational level, smoking status (current/former/never), presence of a stoma, and comorbidities [44] were self-reported at all timepoints.

### 2.5. Statistical Analysis

Descriptive analyses were performed to describe patient characteristics at 6 weeks, overall and by categories of the lifestyle score (with tertiles used as cut-off), and compared to participants for whom a lifestyle score could not be determined (mostly due to the unavailability of accelerometer data). Differences in patient characteristics across the tertiles of lifestyle scores were tested using an ANOVA F test (or Kruskal–Wallis H test for non-normally distributed variables) and Pearson’s χ^2^ test for continuous and categorical variables, respectively. In addition, descriptive analyses were performed for the individual (sub)-recommendations as well as the lifestyle score at all timepoints to describe changes over time. Categorical variables were presented as frequencies with percentages, and for continuous variables, the mean with standard deviation (SD), or medians with interquartile range (IQR), were calculated for normally and non-normally distributed data, respectively. 

Confounder-adjusted linear mixed models were used to assess longitudinal associations of the lifestyle score with HRQoL, fatigue, and CIPN outcomes between 6 weeks and 24 months post-treatment. The lifestyle score was modelled continuously per 1 point increase in level of adherence. We adjusted the regression models for an a priori-defined confounder set, all identified using causal reasoning and based on literature regarding lifestyle and HRQoL in CRC survivors. Regression models were adjusted for fixed (time-invariant) confounders, including age at enrolment (years), sex, education (low, medium, high), and chemotherapy (yes, no), and for time-variant confounders (measured at all post-treatment timepoints), including number of comorbidities (0, 1, ≥2), total energy intake (kcal/day), stoma (yes/no), smoking status (current/former/never), and time since diagnosis (months). In order to gain insight into the influence of each WCRF/AICR recommendation on the lifestyle score, we also performed analyses on the lifestyle score with additional adjustment by the scores for the individual recommendations. The use of random slopes was tested with a likelihood-ratio test; random slopes were added when there was a statistically significant improvement of the model. CIPN outcomes were only analysed for the subgroup of patients who received chemotherapy [45]. The regression coefficients obtained are a weighted average of the between-subject differences and within-person changes. Therefore, separate hybrid models were used to disentangle the intra- and inter-individual components [46]. To estimate the intra-individual associations, individual deviations from the person-mean were modelled to obtain regression coefficients that indicate the changes in HRQoL, fatigue, and CIPN over time due to changes in the lifestyle score within individuals. For the inter-individual associations, centred person-mean values were added to obtain regression coefficients that indicate the difference in HRQoL, fatigue, and CIPN due to differences between individual’s mean levels of the lifestyle score. 

To explore how the components of the lifestyle score (physical activity, body composition, and dietary intake) are independently associated with HRQoL outcomes, fatigue, and CIPN, we performed an analysis with the three subscores in one model, i.e., a physical activity score (0–1), body composition score (0–1), and dietary score (0–5). Since we previously observed that increased alcohol intake was positively related with better HRQoL and less fatigue [27], we assumed that alcohol could possibly be a counteractive component within the dietary score. Therefore, we also performed a post-hoc analysis with the physical activity subscore, body composition subscore, and dietary subscore excluding the alcohol recommendation.

Since there were *n* = 81 participants without a lifestyle score due to missing data on one or more of the individual recommendations (mostly due to unavailability of accelerometer data), a sensitivity analysis was performed to explore the influence of these missing participants. For that purpose, we included the lifestyle score as a relative score in order to include participants with a maximum of one or two missing (sub)scores. For participants with one or two missing (sub)scores, a relative lifestyle score was computed by dividing their available score by the maximum score they would be able to achieve and then multiplied by seven. Lastly, associations for both groups were compared to each other whether the overall conclusions would have been different when accounting for missing values in this way.

Two-sided tests were performed with *p* < 0.05 considered as statistically significant. All statistical analyses were conducted in Stata 15.0 (StataCorp. 2017. College Station, TX, USA).

## 3. Results

Table 2 summarizes the characteristics of study participants at the 6 weeks follow-up time point (*n* = 396) by tertiles of the lifestyle score. Of the total number of participants, 68% were males, the mean age was 67 years (SD: 9.1), and 51% reported two or more comorbid conditions. As expected, there were differences in participant characteristics between the low adherence group for the WCRF/AICR recommendations (first tertile of the lifestyle score) and the high adherence group (third tertile of the lifestyle score). The high adherence group had a notably higher percentage of normal weight participants, reported higher median levels of MVPA, had lower levels of sedentary behaviour, and reported greater intake of fruit, vegetable, and dietary fibre and lower intakes of sugar-sweetened drinks, alcohol, red and processed meat, and UPF compared to the low adherence group. The participants with missing lifestyle scores (20.5%) were mostly similar to the low adherence group and also reported slightly lower quality of life and functioning scores, as well as higher fatigue.

### 3.1. Changes in Level of Adherence and HRQoL, Fatigue, and CIPN Outcomes up to 24 Months Post-Treatment

Across all timepoints, the level of adherence to individual WCRF/AICR recommendations was lowest for red and processed meat (e.g., only 2.9% completely meeting the recommendation at 6 weeks), and second lowest for dietary fibre (e.g., 7.3% complete adherence at 6 weeks). Most participants adhered to the 150 min/week of MVPA recommendation (82.1% at 6 weeks—Table 1). Although participants showed statistically significant changes over time in the individual WCRF/AICR lifestyle recommendations, the mean lifestyle score did not change significantly (*p* = 0.127) and remained, on average, around 3.0 points (SD: 0.8) at all post-treatment timepoints. No participant reached the maximum lifestyle score of 7 at any time point.

On average, HRQoL increased whereas fatigue and CIPN symptoms decreased from 6 weeks up to 24 months post-treatment. The changes over time for HRQoL, fatigue, and CIPN are explained in detail by Kenkhuis and colleagues [24].

### 3.2. Longitudinal Associations of the Lifestyle Score with HRQoL, Fatigue, and CIPN

**Figure 1:**
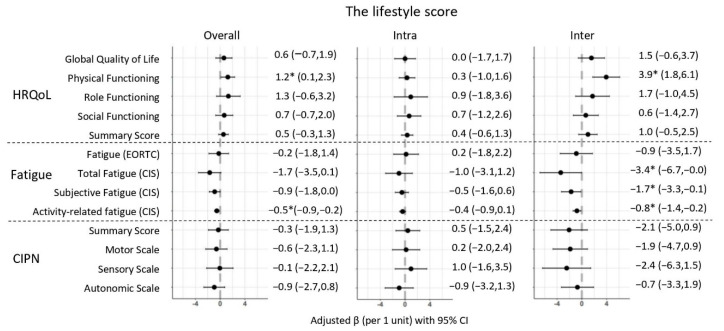
Forest plots showing the confounder-adjusted beta’s (β) and 95% confidence intervals (CI) for the overall longitudinal, intra-, and inter-individual associations of the lifestyle score in relation to health-related quality of life, fatigue, and chemotherapy-induced peripheral neuropathy in stage I-III colorectal cancer survivors followed-up from 6 weeks up to 24 months post-treatment. Model adjusted for sex (male/female), age at enrolment (years), co-morbidities (0, 1, ≥2), education (low, medium, high), chemotherapy (yes/no), total energy intake (kcal/day), stoma (yes/no), smoking status (current/former/never), and time since diagnosis (months). * Indicates statistically significant associations (*p* < 0.05) between the lifestyle score and health-related quality of life, fatigue and CIPN outcomes. Results exploring the influence of the individual recommendations on observed associations for the lifestyle score (Table 3) showed both attenuated and inflated associations for HRQoL and fatigue. We did not explore this in CIPN because no overall associations were found. When additionally adjusted for the physical activity subscore (both MVPA and sedentary behaviour), longitudinal associations attenuated and were all non-significant (e.g., physical functioning and activity-related fatigue). When the lifestyle score was additionally adjusted for the alcohol subscore, however, associations with all HRQoL and fatigue outcomes were inflated and became statistically significant. The lifestyle score additionally adjusted for alcohol was statistically significantly associated with better global QoL (1.9; 0.6,3.3), physical functioning (2.6; 1.4,3.8), role functioning (3.3; 1.3,5.3), and social functioning (2.0; 0.5,3.4), and with less fatigue (EORTC: −1.9; −3.6,−0.2), total fatigue (CIS: −3.7; −5.7,−1.8), subjective fatigue (−1.8; −2.8,−0.9), and activity-related fatigue (−0.9; −1.3,−0.5). Associations of the lifestyle score with HRQoL and fatigue were only slightly attenuated in case of additional adjustment with UPF, sugar-sweetened drinks, and plant-based foods (both fruit and vegetables, and dietary fibre). Slightly inflated associations were seen in the case of additional adjustment for body composition (both BMI and waist circumference). Since the additional adjustment for both the alcohol and body composition recommendation showed inflated results, we therefore decided to also adjust for both. When additionally adjusted for alcohol and body composition, longitudinal associations with better HRQoL and less fatigue were the largest and significant on all subscales.

Figure 1 depicts regression coefficients from confounder-adjusted linear mixed models, representing the overall longitudinal associations from 6 weeks to 24 months post-CRC treatment, as well as intra- and inter-individual associations. In fully-adjusted models, a higher lifestyle score was statistically significantly associated with better physical functioning (β per 1 point: 1.2; 95% CI: 0.1,2.3) and less activity-related fatigue (−0.5; −0.9, −0.2). These associations were also observed in the inter-individual, but not the intra-individual analyses, indicating that a difference in the level of adherence to WCRF/AICR recommendations between individuals, instead of an increase in the level of adherence over time within individuals, was driving the overall associations with better functioning and less fatigue over time. In particular, inter-individual analyses showed that a 1-point difference in the lifestyle score, on average, over time between individuals was associated with better physical functioning (3.9; 1.8,6.1) and less total fatigue (CIS: −3.4; −6.7,−0.0), subjective fatigue (−1.7; −3.3,−0.1), and activity-related fatigue (−0.8; −1.4,−0.2). No overall associations were found for CIPN, but inter-individual analyses showed that a higher lifestyle score was associated with less CIPN complaints on all subscales, although betas were not statistically significant (Figure 1).

An analysis of all components of the lifestyle recommendations included into one model showed the greatest associations for the physical activity component (Table 4). The physical activity subscore, including both MVPA and sedentary behaviour, was statistically significantly associated with all HRQoL and fatigue subscales, independent of the other components. The body composition and dietary intake component were not statistically significantly associated with HRQoL and fatigue. In the model with the physical activity subscore, body composition subscore, and dietary subscore excluding the alcohol recommendation (Table 4), better adherence to the dietary intake component was statistically significantly associated with better physical functioning (β per 1 sub-recommendation: 0.5; 95% CI: 0.1, 0.8) and with less total fatigue (CIS: −0.6; −1.1, −0.0), subjective fatigue (−0.3; −0.5, −0.0), and activity-related fatigue (−0.1; −0.2, −0.0). Furthermore, this additional analysis was not performed with CIPN because no overall associations were observed.

### 3.3. Sensitivity Analyses

When participants with missing data on one or two sub-recommendations were included with a relative score, longitudinal associations were attenuated in comparison to the overall associations including only participants with data on all recommendations, but the direction of the associations remained (Supplementary Table S1). In the analysis with the relative score, the longitudinal association found for activity-related fatigue remained statistically significant.

## 4. Discussion

To the best of our knowledge, this is the first study that assessed longitudinal relationships of a lifestyle score, based on WCRF/AICR recommendations, with HRQoL, fatigue, and CIPN in CRC survivors, from 6 weeks to 24 months post-treatment. In accordance with other studies [17,47], we observed that most participants moderately adhered to several of the individual WCRF/AICR lifestyle recommendations (i.e., BMI, sedentary behaviour, sugary drink consumption, and fruit and vegetable intake). However, adherence was very low for some other recommendations, such as dietary fibre, and red and processed meat consumption, whereas it was very high for the MVPA recommendation. The mean lifestyle score remained similar, on average, across all post-treatment time points (3.1 points), even though changes over time in the mean level of adherence to individual WCRF/AICR lifestyle recommendations were observed. Noteworthy, none of the participants reached the maximum lifestyle score of seven, leaving room for improvement. 

In the confounder-adjusted analysis, we observed that a higher lifestyle score was associated with better physical functioning and less activity-related fatigue. The observed association is consistent with previous studies examining lifestyle scores among CRC survivors [17,19]. Cross-sectional associations have been observed between the number of favourable lifestyle factors (including weight, height, diet, physical activity, and smoking) and physical, role, and social functioning domains [19]. In addition, two cross-sectional studies in CRC survivors reported that higher adherence to a lifestyle score was significantly associated with better HRQoL and with less fatigue [17,18]. A longitudinal study found that a higher WCRF/AICR score at diagnosis was associated with less fatigue at 6 months post-treatment [48]. Our results extend these previous findings by demonstrating similar associations in a longitudinal study in CRC survivors. In addition, observed associations of the lifestyle score with HRQoL and fatigue in our analyses appeared to be mainly driven by between-person differences, pointing towards a role of differences in overall lifestyle between CRC survivors.

Previous research conducted by our own research group found individual associations for HRQoL and fatigue in line with associations for cancer prevention, such as physical activity, sedentary behaviour, fruit and vegetable, dietary fibre, ultra-processed food, red and processed meat, and sugar-sweetened drinks [24,25,26,27]. However, we also previously found associations opposite to the associations found for cancer prevention such as body composition [24] and alcohol [27]. WCRF/AICR recommend having a healthy body weight and limiting alcohol consumption for the prevention of cancer; however, we observed that a higher BMI and increased alcohol consumption in the first 2 years post-treatment were associated with better HRQoL and less fatigue over time in CRC survivors. Thus, in contrast to what is recommended for cancer prevention, increased body composition and increased alcohol consumption may be related to improved HRQoL and reduced fatigue complaints experienced by CRC survivors in the first years after the end of their treatment. These ‘opposing’ associations for body composition and alcohol could be responsible for cancelling out a potential overall effect when making use of the WRF/AICR lifestyle score in relation to these patient-reported outcomes in cancer survivors. In other words, despite not observing (statistically significant) associations for the overall lifestyle score, there are still likely to be independent associations between different lifestyle factors and HRQoL and fatigue, as the previous findings for adherence to the individual recommendations have indicated [24,25,26,27].

Analyses exploring the importance of the individual recommendations on the lifestyle score support this line of thought. Slight or substantial attenuations were observed for the associations for the individual recommendations in line with the cancer prevention recommendations. When separately adjusted for UPF, sugar-sweetened drinks, red and processed meat, and plant-based foods, the longitudinal associations for HRQoL and fatigue slightly attenuated because part of the association was removed by adjusting for it. The substantial attenuation when adjusting for the physical activity subscore, and by MVPA and sedentary behaviour separately, suggests that MVPA and sedentary behaviour are important components of the lifestyle score with regard to associations with HRQoL and fatigue. In contrast, adjustment for the body composition subscore slightly inflated the overall associations, whereas adjustment for the alcohol subscore substantially inflated the overall associations. This means that when included, these components counteracted the overall association of the WCRF/AICR lifestyle score with HRQoL and fatigue. In our previous publication, we suggested that an increased BMI could be a sign of restoration of body composition in the immediate post-treatment recovery period and, therefore, could be positively related to recovery of quality of life and functioning and decreased fatigue and CIPN symptoms [24]. For alcohol consumption, it was suggested that the relationship between alcohol and HRQoL and fatigue outcomes is possibly bidirectional. Participants who start feeling better and less fatigued in the post-treatment period may be more likely to consume alcohol in comparison to people who do not feel better yet [27]. This could be a reason for observing associations of increased alcohol consumption with better HRQoL and less fatigue in CRC survivors, and is probably responsible for our observation that an adjustment for the alcohol subscore inflated associations of the other lifestyle factors with these patient-reported outcomes.

In terms of clinical relevance, the observed effect sizes from the fully-adjusted models were smaller than the minimal clinically important differences [49,50,51]. However, these associations may become larger when adhering to more recommendations, as recommended by WCRF/AICR, who consider their lifestyle recommendations as a package directing people towards a healthy lifestyle and having the largest impact when taken together. Even small changes could already be beneficial, as all small changes accumulate when individuals adhere to more recommendations.

This study has several strengths. An important strength of the current study is the prospective nature and repeated-measures design. Another strength is the use of elaborate measurement instruments (7-day dietary records, objective accelerometers, and extensive anthropometric measurements). Other strengths of our study included the high response rates during follow-up (>90%) and the availability of extensive data on potential confounders. Furthermore, the mixed models enabled the disentangling of inter- and intra-individual associations.

There are also limitations to be considered. Based on the observational data with time-varying exposures and outcomes, we cannot be sure of the direction and potential causality of associations between lifestyle adherence and HRQoL, fatigue, and CIPN. Intervention studies will be necessary to infer causality. In addition, the limited response rate at diagnosis (45%) might have resulted in a selection bias. Participants with less favourable lifestyle conditions and lower HRQoL may have been less likely to participate, and this may have led to an attenuation of associations. Furthermore, the results from the sensitivity analysis may indicate selective missings for the lifestyle score. The participants with missing lifestyle scores were mostly similar to the low adherence group and reported slightly lower HRQoL and more fatigue. Nonetheless, the inclusion of these participants in the form of a relative lifestyle score led to a slight attenuation of the overall associations. A possible explanation might be that by including the relative score, a restriction of range effect could have led to attenuated associations on average. No firm conclusions can be drawn, however. Importantly, the direction of the associations remained. In addition, we cannot rule out the possibility of false positive findings due to the large number of tests performed. Finally, a healthy lifestyle, including a healthy diet, often co-exists with, for instance, better sleep, a larger social network [52], and daily meaningful occupations, and are thus not limited to the seven WCRF/AICR recommendations and three outcomes assessed in this manuscript. Therefore, the inter-individual associations found might also be explained by residual lifestyle factors not taken into account in the lifestyle score.

Next to the advantages of using a lifestyle score (e.g., lifestyle is considered to be a package of correlated unhealthy and healthy habits), there are also some disadvantages to using the lifestyle score. Overall, it is a simple overall scoring system, weighing all recommendations equally under the assumption that each lifestyle behaviour has an equal impact on HRQoL, fatigue, or CIPN, and hence disregarding that some recommendations might be of greater importance than others (e.g., physical activity). In addition, possible interactions between individual lifestyle recommendations in relation to outcomes, as observed for MVPA and sedentary behaviour in relation to HRQoL and fatigue by Kenkhuis and colleagues, [25], are also disregarded. Moreover, there are five dietary components within the lifestyle score, giving greater weight to diet than body composition and physical activity. Besides these disadvantages of the lifestyle score, which were also previously addressed by Shams-White and colleagues [28], the opposing associations found of alcohol and body composition with HRQoL and fatigue [24,36] point towards additional challenges. These challenges may raise questions as to whether all lifestyle recommendations are equally applicable to patient-reported outcomes, such as HRQoL, that differ from clinical or prognostic outcomes, such as cancer development, recurrence, and mortality. All the above-mentioned disadvantages underscore the difficulty of investigating the recommendations as a package by means of the lifestyle score, as suggested by WCRF and AICR, possibly resulting in not observing any associations or mitigated associations. It needs to be emphasized, however, that not finding an overall association for the lifestyle score does not mean that promoting a healthy lifestyle through the recommendations is not relevant. It merely emphasizes the importance of a cautious interpretation when using a lifestyle score for investigating the role of lifestyle as a whole, especially in relation to more subjective patient-reported outcomes such as HRQoL, fatigue, and CIPN.

## 5. Conclusions

In conclusion, the results from this study add to the existing body of literature on lifestyle and HRQoL, fatigue, and CIPN. Altogether, our results show that there is ample room for improvement of the lifestyle of CRC survivors. Moreover, in this study, we observed that adhering to more WCRF/AICR recommendations regarding a healthy lifestyle was associated with better physical functioning and less activity-related fatigue. Studying lifestyle behaviours as a whole, and changes therein by means of operationalizing these behaviours in the form of a lifestyle score, is challenging, especially in HRQoL research where opposing associations for individual lifestyle recommendations may complicate the interpretation of an overall lifestyle score. Nevertheless, the longitudinal associations of the WCRF/AICR lifestyle score with HRQoL and fatigue observed in our study suggest that there is potential for lifestyle interventions for CRC survivors in the years after the end of their treatment. Such lifestyle interventions would ideally be multifactorial, directed at the improvement of physical activity and diet quality to enhance HRQoL and decrease fatigue symptoms.

## Figures and Tables

**Table 1:** Adherence to the 2018 WCRF/AICR recommendations [12] by colorectal cancer survivors from 6 weeks to 24 months post-treatment.

2018 WCRF/AICR Recommendations		Operationalization of Recommendations	Adherence to the Individual Recommendations				
			Points	6 Weeks *n* (%) ^a^	6 Months *n* (%) ^a^	12 Months *n* (%) ^a^	24 Months *n* (%) ^a^
**1.**	Be a healthy weight	BMI (kg/m^2^):					
		18.5–24.9 25–29.9 <18.5 or ≥30	0.5 0.25 0	116 (29.5) 172 (43.8) 105 (26.7)	89 (25.7) 151 (43.6) 106 (30.6)	61 (21.6) 130 (46.1) 91 (32.3)	49 (24.0) 85 (41.7) 70 (34.3)
		Waist circumference (cm):					
		Men: <94 or women: <80 Men: 94- < 102 or women: 80- < 88 Men: ≥102 or women: ≥88	0.5 0.25 0	87 (22.0) 88 (22.3) 220 (55.7)	61 (17.6) 85 (24.5) 201 (57.9)	49 (17.2) 62 (21.8) 174 (61.1)	41 (20.0) 39 (19.0) 125 (61.0)
**2.**	Be physically active	Total self-reported moderate-vigorous physical activity (min/wk):					
		≥150 75- < 150 <75	0.5 0.25 0	320 (82.1) 28 (7.2) 42 (10.8)	302 (87.5) 13 (3.8) 30 (8.7)	255 (90.1) 11 (3.9) 17 (6.0)	181 (90.5) 8 (4.0) 11 (5.5)
		Accelerometer-assessed prolonged sedentary behavior (h/day):					
		≤3 >3–6 >6	0.5 0.25 0	70 (21.5) 148 (45.5) 107 (32.9)	85 (29.3) 154 (53.1) 51 (17.6)	58 (24.8) 126 (53.9) 50 (21.4)	45 (25.7) 93 (53.1) 37 (21.1)
**3.**	Eat a diet rich in wholegrains, vegetables, fruit and beans	Fruits and vegetables (g/day):					
		≥400 200- < 400 <200	0.5 0.25 0	44 (11.5) 198 (51.7) 141 (36.8)	45 (13.5) 146 (43.8) 142 (42.6)	38 (13.9) 123 (44.9) 113 (41.2)	28 (14.4) 88 (45.4) 78 (40.2)
		Total dietary fiber (g/day):					
		≥30 15- < 30 <15	0.5 0.25 0	28 (7.3) 298 (77.8) 57 (14.9)	21 (6.3) 263 (79.0) 49 (14.7)	19 (6.9) 208 (75.9) 47 (17.2)	13 (6.7) 143 (73.7) 38 (19.6)
**4.**	Limit consumption of “fast foods” and other processed foods high in fat, starches or sugars	Percent of total kcal from ultra-processed foods (UPFs):					
		Tertile 1 Tertile 2 Tertile 3	1 0.5 0	128 (33.5) 127 (33.3) 127 (33.3)	120 (36.0) 121 (36.3) 92 (27.6)	102 (37.2) 101 (36.9) 71 (25.9)	78 (40.2) 66 (34.0) 50 (25.8)
**5.**	Limit consumption of red and processed meat	Total red meat (g/wk) and processed meat (g/wk):					
		Red meat < 500 and processed meat < 21 Red meat < 500 and processed meat 21-<100 Red meat > 500 or processed meat ≥ 100	1 0.5 0	11 (2.9) 23 (6.0) 349 (91.1)	9 (2.7) 25 (7.5) 299 (89.8)	11 (4.0) 22 (8.0)241 (88.0)	9 (4.6) 14 (7.2) 171 (88.1)
**6.**	Limit consumption of sugar-sweetened drinks	Total sugar-sweetened drinks (g/day):					
		0 >0- ≤ 250 >250	1 0.5 0	67 (17.5) 248 (64.8) 68 (17.8)	71 (21.3) 218 (65.5) 44 (13.2)	71 (25.9) 173 (63.1) 30 (11.0)	44 (22.7) 126 (65.0) 24 (12.4)
**7.**	Limit alcohol consumption	Total ethanol (g/day):					
		0 >0- ≤ 10 >10	1 0.5 0	122 (31.9) 98 (25.6) 163 (42.6)	104 (31.2) 84 (25.2) 145 (43.5)	75 (27.4) 72 (26.3) 127 (46.4)	58 (29.9) 49 (25.3) 87 (44.9)
**The lifestyle score**^b^—Mean (SD) Range			0–7	3.0 (0.8) 0.75–5.5	3.1 (0.8) 0.5–5.75	3.1 (0.8) 1–5.75	3.1 (0.8) 1.25–5.25

BMI. body mass index; EN%. Energy percentage; g. gram; h. hours; SD. standard deviation; UPF. ultra-processed foods; wk. week. ^a^ Percentages may not add up to 100 due to rounding. ^b^ For the lifestyle score there were 81 missings at 6 weeks, 66 missings at 6 months, 61 misings at 12 months, and 37 missings at 24 months. Missings were mostly due to missing accelerometer data. Sedentary behaviour was missing for 71 participants at 6 weeks, 58 participants at 6 months, 53 participants at 12 months, and 33 participants at 24 months. Across timepoints, principally the same participants had missing accelerometer data, due to unwillingness to wear the accelerometer. This was not the case for other missings. For the dietary recommendations, there were 13 missings at 6 weeks, 15 missings at 6 months, 13 missings at 12 months, and 14 missings at 24 months. Missings from accelerometer data and dietary data may not add up because participants can also miss both.

**Table 2:** Demographic, lifestyle, and clinical characteristics of colorectal cancer survivors, by categories of the lifestyle score (with tertiles used as cut-off) and compared to participants who did not have a lifestyle score at 6 weeks.

	All Participants at 6 Weeks Post-Treatment (*n* = 396) ^a^	1 Tertile Lifestyle Score < 2.75 (*n* = 102) ^a^	2nd Tertile Lifestyle Score 2.75–3.5 (*n* = 107) ^a^	3rd Tertile Lifestyle Score > 3.5 (*n* = 106) ^a^	*p*-Value ^c^	Missing Lifestyle Score (*n* = 81) ^a^
Socio-demographic						
Sex (male) [n (%)]	270 (68.2)	75 (73.5)	66 (61.7)	71 (67.0)	0.19	58 (71.6)
Age (years) [mean (SD)]	67.0 (9.1)	66.7 (9.3)	65.9 (8.5)	68.4 (8.7)	0.62	67.2 (10.0)
Comorbidities					0.15	
0	91 (23.0)	19 (18.6)	26 (24.3)	23 (21.7)		23 (28.8)
1	102 (25.8)	24 (23.5)	36 (33.6)	24 (22.6)		18 (22.5)
≥2	202 (51.1)	59 (57.8)	45 (42.1)	59 (55.7)		39 (48.8)
Education [n (%)]					0.36	
Low	107 (27.1)	29 (28.4)	25 (23.4)	30 (28.3)		23 (28.8)
Medium	149 (37.7)	36 (35.3)	51 (47.7)	38 (35.9)		24 (30.0)
High	139 (35.2)	37 (36.3)	31 (29.0)	38 (35.9)		33 (41.3)
Clinical						
Cancer type [n (%)]					0.80	
Colon	250 (63.1)	66 (64.7)	70 (65.4)	65 (61.3)		49 (60.5)
Rectosigmoid and rectum	146 (36.9)	36 (35.3)	37 (34.6)	41 (38.7)		32 (39.5)
Tumour stage [n (%)]					0.17	
Stage I	124 (31.3)	41 (40.2)	32 (29.9)	31 (29.3)		20 (24.7)
Stage II	100 (25.3)	18 (17.7)	28 (26.2)	33 (31.1)		21 (25.9)
Stage III	172 (43.4)	43 (42.2)	47 (43.9)	42 (39.6)		40 (49.4)
Treatment [n (%)]						
Surgery (yes)	354 (89.4)	85 (83.3)	97 (90.7)	95 (89.6)	0.22	77 (95.1)
Chemotherapy (yes)	155 (39.1)	35 (34.3)	43 (40.2)	36 (34.0)	0.57	41 (50.6)
Radiotherapy (yes)	101 (25.5)	20 (19.6)	28 (26.2)	26 (24.5)	0.51	27 (33.3)
Stoma (yes)	110 (28.4)	29 (28.4)	30 (28.0)	26 (24.8)	0.81	25 (33.8)
Lifestyle						
Smoking [n (%)]					0.59	
Never	118 (30.5)	27 (26.5)	30 (28.0)	35 (33.3)		26 (35.6)
Former	235 (60.7)	65 (63.7)	70 (65.4)	59 (56.2)		41 (56.2)
Current	34 (8.8)	10 (9.8)	7 (6.5)	11 (10.5)		6 (8.2)
Body mass index, kg/m^2^ [mean (SD)]	27.8 (4.6)	29.1 (4.2)	27.4 (4.1)	26.1 (4.7)	0.39	28.8 (4.7)
Underweight &Healthy weight: <25	119 (31.1)	16 (15.7)	30 (28.0)	52 (49.1)	0.00 *	21 (26.3)
Overweight: 25–29.9	173 (43.8)	47 (46.1)	52 (48.6)	41 (38.7)		33 (41.3)
Obese: ≥30	103 (26.1)	39 (38.2)	25 (23.4)	13 (12.3)		26 (32.5)
Waist circumference (cm) [mean (SD)]	100.1 (12.9)	104.9 (12.5)	98.8 (11.7)	95.4 (12.3)	0.76	101.9 (13.6)
MVPA (min/w) [median (IQR)	7 (10.8)	6 (8.2)	7.5 (12.5)	8.8 (11.0)	0.00 *	6.3 (9.6)
Prolonged sedentary behaviour (h/d) [mean (SD)] ^b^	5.3 (2.7) * (*n* = 325)	5.9 (2.8)	5.1 (2.4)	5.0 (2.9)	0.14	5.9 (1.9) (*n* = 10)
Fruit & vegetables (g/d) [mean (SD)]	251.0 (124.7)	182.5 (96.3)	258.9 (115.6)	320.3 (133.6)	0.01 *	233.2 (101.9)
Dietary fibre (g/d) [mean (SD)]	20.9 (5.8)	18.2 (4.6)	21.4 (5.3)	23.7 (6.6)	0.00 *	19.9 (4.9)
Sugary drinks (g/d) [mean (SD)]	133.7 (157.9)	188.6 (201.4)	133.5 (157.2)	95.3 (114.6)	0.00 *	111.4 (117.8)
Alcohol (g/d) [mean (SD)]	13.1 (19.1)	15.0 (14.3)	10.8 (16.0)	10.6 (19.5)	0.01 *	17.8 (26.9)
Red meat (g/w) [mean (SD)]	593.4 (294.8)	634.3 (303.8)	629.6 (294.2)	529.3 (292.6)	0.92	574.9 (271.4)
Processed meat (g/w) [mean (SD)]	325.1 (209.4)	399.4 (225.9)	324.7 (194.4)	272.2 (199.4)	0.26	296.7 (193.2)
Ultra-processed food (EN%) [mean (SD)]	35.4 (10.8)	40.4 (8.8)	36.6 (11.2)	28.8 (9.6)	0.04 *	36.2 (9.6)

BMI. body mass index; d. day; EN%. energy percentage; g. gram; h. hours; SD. standard deviation; w. week. ^a^ Percentages may not add up to 100 due to rounding. ^b^ Only 10 participants had MOX data available to assess sedentary behaviour in the group missing the lifestyle score. ^c^ Differences in patient characteristics across the tertiles of lifestyle scores. * Indicates statistically significant associations (*p* < 0.05) between the lifestyle score and health-related quality of life, and fatigue outcomes.

**Table 3:** Linear mixed models between the lifestyle score in relation to health-related quality of life and fatigue, both overall and adjusted for the individual WCRF/AICR lifestyle recommendation subscores.

	EORTC QLQ-C30						Checklist Individual Strength		
	Global QoL (0–100)	Physical Functioning (0–100)	Role Functioning (0–100)	Social Functioning (0–100)	Summary Score (0–100)	Fatigue (EORTC) (0–100)	Fatigue (CIS) (20–140)	Subjective Fatigue (CIS) (8–56)	Activity Fatigue (CIS) (3–21)
	β (95% CI) ^b^	β (95% CI)	β (95% CI)	β (95% CI)	β (95% CI)	β (95% CI)	β (95% CI)	β (95% CI)	β (95% CI)
Lifestyle score (per 1 point increase)									
Adjusted ^a^	0.6 (−0.7,1.9)	1.2 * (0.1,2.3)	1.3 (−0.6,3.2)	0.7 (−0.7,2.0)	0.5 (−0.3,1.3)	−0.2 (−1.8,1.4)	−1.7 (−3.5,0.1)	−0.9 (−1.8,0.0)	−0.5 * (−0.9,−0.2)
Additional adjustment for:									
Physical activity ^c^	−0.5 (−1.8,0.9)	0.3 (−0.9,1.4)	−0.5 (−2.5,1.4)	−0.3 (−1.7,1.2)	−0.1 (−0.9,0.7)	0.9 (−0.8,2.5)	−0.3 (−2.1,1.6)	−0.2 (−1.1,0.7)	−0.2 (−0.6,0.2)
MVPA	−0.0 (−1.3,1.3)	0.7 (−0.4,1.8)	0.1 (−1.8,2.0)	0.1 (−1.3,1.5)	0.2 (−0.6,1.0)	0.3 (−1.3,1.9)	−1.0 (−2.9,0.8)	−0.5 (−1.4,0.4)	−0.4 * (−0.8,−0.0)
Sedentary behaviour	0.1 (−1.3,1.4)	0.7 (−0.4,1.8)	0.5 (−1.5,2.5)	0.2 (−1.2,1.6)	0.2 (−0.6,1.0)	0.5 (−1.2,2.1)	−0.8 (−2.6,1.1)	−0.6 (−1.5,0.3)	−0.1 (−0.5,0.4)
Body composition ^d^	0.6 (−0.8,2.0)	1.5 * (0.3,2.6)	1.4 (−0.6,3.5)	0.8 (−0.7,2.3)	0.6 (−0.2,1.4)	0.0 (−1.7,1.6)	−1.6 (−3.5,0.4)	−0.8 (−1.7,0.2)	−0.5 * (−0.9,−0.1)
Plant-based foods ^e^	0.6 (−1.0,2.1)	1.0 (−0.3,2.3)	1.4 (−0.9,3.6)	0.6 (−1.1,2.2)	0.3 (−0.6,1.2)	−0.3 (−2.1,1.6)	−1.6 (−3.7,0.5)	−0.8 (−1.8,0.3)	−0.5 * (−1.0,−0.1)
Ultra-processed foods	0.4 (−1.2,2.0)	1.0 (−0.3,2.4)	0.8 (−1.5,3.1)	−0.1 (−1.8,1.5)	0.1 (−0.8,1.1)	0.5 (−1.4,2.4)	−1.4 (−3.6,0.8)	−0.8 (−1.8,0.3)	−0.5 * (−1.0,0.1)
Red and processed meat	0.6 (−0.8,2.0)	1.2 * (0.0,2.4)	1.5 (−0.5,3.5)	0.8 (−0.7,2.2)	0.7 (−0.2,1.5)	−0.6 (−2.3,1.1)	−2.2 (−4.1,−0.3)	−1.1 (−2.0,−0.2)	−0.6 * (−1.0,−0.1)
Sugar-sweetened drinks	0.4 (−1.1,1.8)	0.9 (−0.3,2.1)	0.7 (−1.4,2.8)	0.2 (−1.3,1.8)	0.2 (−0.7,1.1)	0.2 (−1.5,2.0)	−0.9 (−2.9,1.1)	−0.7 (−1.6,0.3)	−0.5 * (−0.9,−0.0)
Alcohol	1.9 * (0.5,3.3)	2.6 * (1.4,3.8)	3.3 * (1.3,5.3)	2.0 * (0.5,3.4)	1.7 * (0.8,2.5)	−1.9 * (−3.6,−0.2)	−3.7* (−5.7,−1.8)	−1.8 * (−2.8,−0.9)	−0.9 * (−1.3,−0.5)
Body composition and alcohol	2.1 * (0.6,3.5)	3.0 * (1.7,4.2)	3.7 * (1.5,5.8)	2.3 * (0.8,3.9)	1.8 * (0.9,2.7)	−1.9 * (−3.6,−0.1)	−3.7 * (−5.8,−1.7)	−1.7 * (−2.7,−0.8)	−0.9 * (−1.3,−0.5)

Abbreviations: EORTC QLQ-C30, European Organization for the Research and Treatment of Cancer Quality of Life; β, beta-coefficient; CI, confidence interval; Qol, Quality of life. ^a^ Model adjusted for sex (male/female), age enrolment (years), co-morbidities (0, 1, ≥2), education (low, medium, high), chemotherapy (yes/no), total energy intake (kcal/day), stoma (yes/no), smoking (current/former/never), and time since diagnosis (months). ^b^ The beta-coefficients represent the overall longitudinal difference in the outcome score. ^c^ Physical activity score includes both moderate-to-vigorous physical activity and sedentary behaviour components. ^d^ Body composition score includes both BMI and waist circumference components. ^e^ Plant-based foods score includes both fruit and vegetable, and dietary fibre components. * Indicates statistically significant associations (*p* < 0.05) between the lifestyle score and health-related quality of life, and fatigue outcomes.

**Table 4:** Linear mixed models between the physical activity subscore, body composition subscore, and dietary subscore (with and without alcohol), all in one model, in relation to health-related quality of life, and fatigue.

	EORTC QLQ-C30						Checklist Individual Strength		
	Global QOL (0–100)	Physical Functioning (0–100)	Role Functioning (0–100)	Social Functioning (0–100)	Summary Score (0–100)	Fatigue (EORTC) (0–100)	Fatigue (CIS) (20–140)	Subjective Fatigue (CIS) (8–56)	Activity Fatigue (CIS) (3–21)
	β (95% CI) ^b^	β (95% CI)	β (95% CI)	β (95% CI)	β (95% CI)	β (95% CI)	β (95% CI)	β (95% CI)	β (95% CI)
Model with the three subscores including alcohol in the dietary subscore									
Physical activity subscore ^a,c^	3.3 * (2.2,4.4)	3.3 * (2.5,4.2)	5.5 * (3.9,7.1)	2.7 * (1.6, 3.9)	2.0 * (1.4,2.7)	−3.2 * (−4.5,−1.9)	−4.5 * (−6.0,−3.0)	−2.1 * (−2.9,−1.4)	−1.2 * (−1.5,−0.9)
Body composition subscore ^a,d^	−0.0 (−1.0,1.0)	−0.6 (−1.5,0.3)	−0.2 (−1.5,1.1)	−0.2 (−1.2,0.7)	−0.1 (−0.7,0.6)	−0.3 (−1.5,1.0)	−0.5 (−2.0,0.9)	−0.4 (−1.1,0.3)	−0.2 (−0.5,0.1)
Dietary subscore ^a,e^	−0.1 (−0.5,0.2)	0.1 (−0.2,0.4)	−0.1 (−0.7,0.4)	−0.0 (−0.4,0.3)	−0.0 (−0.2,0.2)	0.3 (−0.2,0.7)	−0.0 (−0.5,0.5)	−0.0 (−0.3,0.2)	−0.0 (−0.1,0.1)
Model with the three subscores excluding alcohol in the dietary subscore									
Physical activity subscore ^a,c^	3.3 * (2.2,4.4)	3.3 * (2.4,4.2)	5.4 * (3.8,7.0)	2.7 * (1.6, 3.9)	2.0 * (1.4,2.7)	−3.2 * (−4.5,−1.9)	−4.5 * (−6.0,−3.0)	−2.1 * (−2.8,−1.4)	−1.2 * (−1.5,−0.8)
Body composition subscore ^a,d^	−0.0 (−1.0,1.0)	−0.5 (−1.4,0.4)	−0.2 (−1.5,1.1)	−0.2 (−1.2,0.8)	−0.1 (−0.7,0.6)	−0.3 (−1.5,0.9)	−0.6 (−2.1,0.9)	−0.5 (−1.2,0.2)	−0.2 (−0.5,0.1)
Dietary subscore without alcohol ^a,f^	0.2 (−0.2,0.6)	0.5 * (0.1,0.8)	0.5 (−0.1,1.0)	0.4 (−0.0,0.8)	0.3 * (0.1,0.5)	−0.2 (−0.7,0.2)	−0.6 * (−1.1,−0.0)	−0.3 * (−0.5,−0.0)	−0.1 * (−0.2,−0.0)

Abbreviations: EORTC QLQ-C30, European Organization for the Research and Treatment of Cancer Quality of Life; β, beta-coefficient; CI, confidence interval; Qol, quality of life. ^a^ Model adjusted for sex (male/female), age at enrolment (years), co-morbidities (0, 1, ≥2), education (low, medium, high), chemotherapy (yes/no), total energy intake (kcal/day), stoma (yes/no), smoking (current/former/never), and time since diagnosis (months). ^b^ The beta-coefficients represent the overall longitudinal difference in the outcome score. ^c^ Physical activity subscore includes both moderate-to-vigorous physical activity and sedentary behaviour components. ^d^ Body composition subscore includes both BMI and waist circumference components. ^e^ Dietary subscore includes fruit and vegetable, dietary fibre, ultra-processed food, red and processed meat, and sugar-sweetened drinks and alcohol. ^f^ Dietary subscore without alcohol includes fruit and vegetable, dietary fibre, ultra-processed food, red and processed meat, and sugar-sweetened drinks. * Indicates statistically significant association (*p* < 0.05) between the lifestyle subscores and health-related quality of life, and fatigue outcomes.

## Data Availability

Data described in the manuscript, code book, and analytic code will be made available upon request pending (e.g., application and approval, payment, other). Requests for data of the EnCoRe study can be sent to Martijn Bours, Department of Epidemiology, GROW-School for Oncology and Developmental Biology, Maastricht University, the Netherlands (email: m.bours@maastrichtuniversity.nl).

## Supplementary Materials

The following are available online at https://www.mdpi.com/article/10.3390/cancers14020417/s1, Table S1: Sensitivity analysis with relative lifestyle scores.
