# Supplementary material for: Longitudinal Associations of Adherence to the World Cancer Research Fund/American Institute for Cancer Research (WCRF/AICR) Lifestyle Recommendations with Quality of Life and Symptoms in Colorectal Cancer Survivors up to 24 Months Post-Treatment
Source: Cancers (Basel). 2022 Jan 14;14(2):417. doi: 10.3390/cancers14020417 (PMC8774035; doi:10.3390/cancers14020417)
Supplement: Supplementary file 1 [file cancers-14-00417-s001.zip › cancers-1504908-supplementary.pdf]

Supplementary Table S1: Sensitivity analysis with relative lifestyle scores.

Abbreviations: EORTC QLQ-C30, European Organization for the Research and Treatment of Cancer Quality of Life; β, beta-coefficient; CI, confidence interval; Qol, Quality of life.

	EORTC QLQ-C30						Checklist individual strength		
	Global QOL (0-100)	Physical functioning (0-100)	Role functioning (0-100)	Social functioning (0-100)	Summary score (0-100)	Fatigue (EORTC) (0-100)	Fatigue (CIS) (20-140)	Subjective fatigue (CIS) (8-56)	Activity fatigue (CIS) (3-21)
	β (95% CI)[a,b]	β (95% CI)	β (95% CI)	β (95% CI)	β (95% CI)	β (95% CI)	β (95% CI)	β (95% CI)	β (95% CI)
Participants with all items	0.6 (-0.7,1.9)	1.2* (0.1,2.3)	1.3 (-0.6,3.2)	0.7 (-0.7,2.0)	0.5 (-0.3,1.3)	-0.2 (-1.8,1.4)	-1.7 (-3.5,0.1)	-0.9 (-1.8,0.0)	-0.5* (-0.9,-0.2)
All participants including participants with one or two missing items	0.3 (-1.0,1.5)	0.8 (-0.2,1.9)	0.5 (-1.3,2.3)	0.5 (-0.8,1.9)	0.4 (-0.3,1.2)	0.1 (-1.5,1.6)	-1.0 (-2.7,0.7)	-0.5 (-1.3,0.4)	-0.5* (-0.8,-0.1)

[a] Model adjusted for sex (male/female), age enrolment (years), co-morbidities (0, 1, ≥2), education (low, medium, high), chemotherapy (yes/no), total energy intake (kcal/day), stoma (yes/no), smoking (current/former/never), and time since diagnosis (months).
[b] The beta-coefficients represent the overall longitudinal difference in the outcome score.
